# Association between smoking history and optical coherence tomography angiography findings in diabetic patients without diabetic retinopathy

**DOI:** 10.1371/journal.pone.0253928

**Published:** 2021-07-09

**Authors:** Dong-Wei Liu, Zeeshan Haq, Daphne Yang, Jay M. Stewart

**Affiliations:** 1 Department of Ophthalmology, University of California, San Francisco, San Francisco, California, United States of America; 2 Department of Ophthalmology, Zuckerberg San Francisco General Hospital and Trauma Center, San Francisco, California, United States of America; 3 Department of Ophthalmology, The Second Affiliated Hospital of Anhui Medical University, Hefei, Anhui, China; Massachusetts Eye & Ear Infirmary, Harvard Medical School, UNITED STATES

## Abstract

**Purpose:**

To investigate any associations between cigarette smoking and retinal microvascular changes in diabetic patients without visible retinopathy.

**Design:**

Retrospective, cross-sectional study.

**Participants:**

1099 eyes from 1099 diabetic patients with no clinical evidence of diabetic retinopathy (DR) were included in this study.

**Methods:**

Diabetic patients underwent optical coherence tomography angiography (OCTA) scanning at Zuckerberg San Francisco General Hospital and Trauma Center between April 2018 and September 2019. Patient demographic and clinical information was collected. Standard bivariate statistics and multivariate linear regression were performed.

**Main outcome measures:**

OCTA parameters included metrics related to the foveal avascular zone (FAZ; area, perimeter, circularity), perfusion density (PD; full, center, inner), and vessel length density (VLD; full, center, inner).

**Results:**

The study population included 750 non-smokers and 349 smokers. FAZ perimeter was the only OCTA parameter that was significantly different between the two groups on uncontrolled analysis (P = 0.033). Multivariate regression analyses revealed significant associations between lower VLD full (*β* = -0.31, *P* = 0.048), lower VLD inner (*β* = -0.35, *P* = 0.046) and a history of smoking. No significant associations between cigarette smoking and either FAZ or PD were detected.

**Conclusions:**

Our results suggest that smoking is likely associated with deleterious changes in the retinal microvasculature of patients with a history of diabetes and no visible DR. Based on these findings, diabetic patients with a history of smoking may benefit from higher prioritization in terms of ophthalmic screening.

## Introduction

Diabetes mellitus (DM) leads to microvascular and macrovascular complications that place a heavy burden on societies around the world [1]. One such complication is diabetic retinopathy (DR), which is becoming the leading cause of preventable blindness in working-age populations [2]. DR is a complex, chronic disease caused by interactions between genetic factors and environmental conditions. Implicated risk factors for DR development and progression include age, gender, race, duration of diabetes, hyperglycemia, hypertension, hyperlipidemia, body mass index, and insulin use [3–5]. However, the relationship between smoking and DR remains unclear despite the known association between smoking and other vasculopathic diseases. The epidemiologic literature has reported conflicting results on the specific association of smoking with DR, with some studies finding a higher risk of DR [6] and others showing no effect or even protection against DR [7–10].

For many years, fluorescein angiography (FA) has been considered the gold standard for assessing microvascular changes in retinal vascular diseases such as DR. However, optical coherence tomography angiography (OCTA) has recently emerged as a noninvasive alternative technology. It can visualize blood flow in retinal blood vessels and determine the size of capillaries by detecting the contrast of blood cells in motion. Compared with conventional FA, it is a faster and safer imaging technique with high contrast and resolution [11–14]. Recent work has found widefield swept-source OCTA to be comparable to ultra-widefield fluorescein angiography in the detection of DR lesions [15]. In addition, OCTA can detect preclinical retinal microvascular abnormalities before the appearance of visible microaneurysms [16].

Given the ability of OCTA to detect early diabetes-related vascular changes in the retina, this technology offers a unique opportunity to study associations between smoking and incipient DR development. Prior epidemiologic studies have depended upon the visualization of later-stage clinical manifestations [6–10]. In this setting, the cumulative contribution of other, more determinative, risk factors might have rendered a true association with smoking difficult to confirm. In contrast, a more sensitive OCTA-based approach in patients with no visible DR may be able to identify smaller sub-clinical changes.

At this time, there is a paucity of studies that have examined the relationship between smoking and OCTA-based vascular parameters. One report compared OCTA findings between a small group of smokers and non-smokers and found no differences in central macular perfusion; however, choroidal thickness was larger in the group of smokers [17]. In addition, studies that focus on acute changes immediately after smoking have shown mixed results [18,19]. As such, the relationship between smoking and microvascular changes detected by OCTA remains unclear. The evaluation of patients with DM and no visible DR using OCTA offers an opportunity to quantify discrete changes associated with smoking. Such changes may confer a higher risk of progression to DR, given that microvascular alterations on OCTA can be predictive of subsequent retinopathy risk [20]. For these reasons, we sought to test the hypothesis that smoking is associated with pathologic OCTA-based vascular changes in patients with DM and no visible DR.

## Materials and methods

### Participants

This retrospective cross-sectional study involved patients evaluated through a DR screening program at Zuckerberg San Francisco General Hospital and Trauma Center from April 2018 to September 2019. This study was approved by the Human Research Protection Program at the University of California, San Francisco and followed the tenets of the Declaration of Helsinki.

Adult (18 years or older) patients with type 1 or type 2 DM presenting for DR screening underwent ultra-widefield fundus photography (Optos Daytona, Optos PLC, Dunfermline, UK) and OCTA imaging during the visit. All eyes without detectable DR on the ultra-widefield fundus images were considered for inclusion in the study. Eyes with a history of ocular trauma or any form of ocular disease, with the exception of cataract, were excluded.

### Clinical data

Collected information included age, sex, race, duration of diabetes mellitus, most recent hemoglobin A1c level (HbA1c), insulin use, body mass index (BMI), hypertension, hyperlipidemia, and smoking history. Smoking history was self-reported by the patient through solicitation at the DR screening visit [21,22]. The smoking history was used to classify patients into two categories. Patients who had never smoked or had smoked fewer than 100 cigarettes in their lifetime [23] were labeled as non-smokers. Patients who had smoked greater than 100 cigarettes in their lifetime irrespective of current smoking status were labeled as smokers.

### OCTA image acquisition

OCTA imaging was obtained using a Cirrus^™^ HD-OCT 5000 with AngioPlex OCT Angiography (Carl Zeiss Meditec, Dublin, CA, USA) machine. A 3 x 3 mm scan area centered on the fovea was obtained in the right eye and then the left eye of each patient. Each scan included 245 clusters of B-scans repeated at least twice for repeatability analysis, and each B-scan consisted of 245 A-scans. FastTrac motion-correction software was used during image acquisition to reduce artifact. Images were considered to be of satisfactory quality by the following criteria: signal strength (SS) of greater than or equal to 7, maintenance of the fovea within the center of the scanned image without significant decentration, minimal blinking, and no more than one motion artifact.

### OCTA image processing

The following parameters were generated automatically by the built-in OCTA analysis software (CIRRUS 11.0; clearance by the US Food and Drug Administration pending): foveal avascular zone (FAZ), perfusion density (PD), and vessel length density (VLD) in the superficial capillary plexus (SCP). The SCP was defined by an inner boundary at the internal limiting membrane and an outer boundary at the junction of the inner plexiform layer and the inner nuclear layer. For each patient, the images for each eye were assessed for artifactual errors and the eye with fewer errors was selected for analysis. If such errors were similar, the eye with the higher SS was selected. If both quality metrics were similar, the right eye was selected.

**Fig 1:**
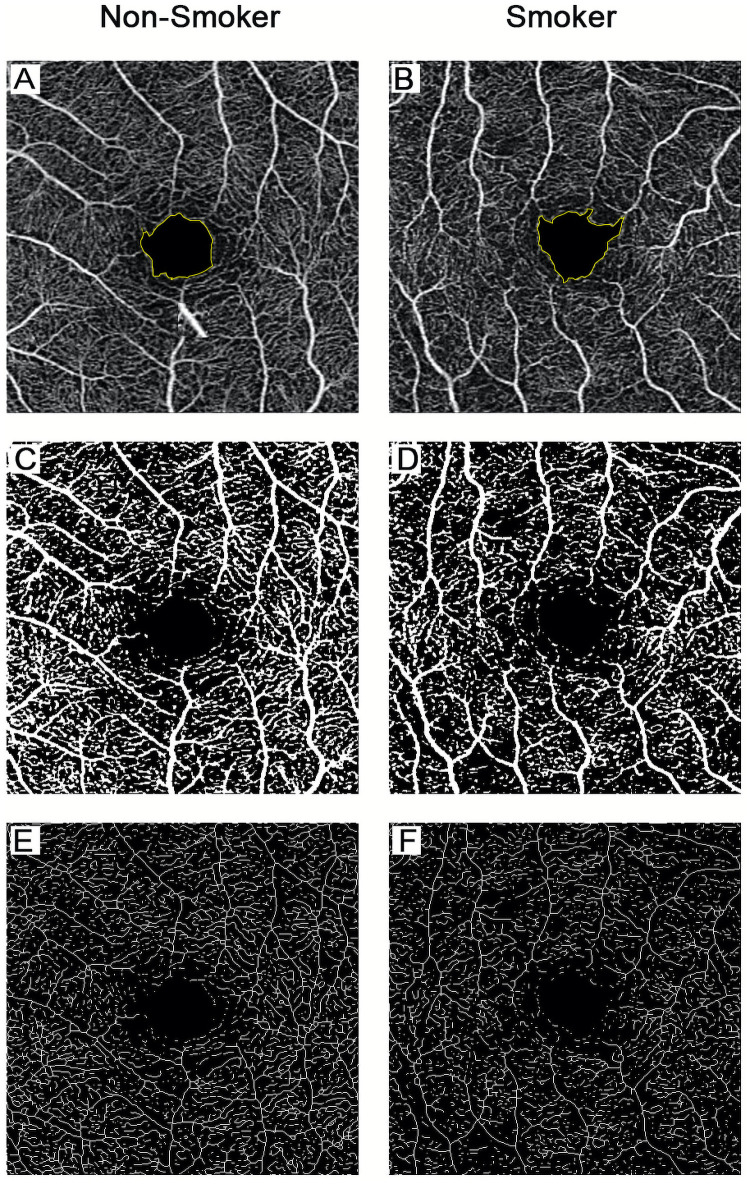
Optical coherence tomography (OCTA) image acquisition from SCP for each group. The FAZ was automatically delineated by the software (A, B). The perfused vasculature was automatically segmented by the software to calculate PD (C, D). The skeletonized vasculature was automatically segmented by the software to calculate VLD (E, F). Each column shows a representative subject from each group of participants. SCP = superficial capillary plexus, FAZ = foveal avascular zone, PD = perfusion density, VLD = vessel length density.

**Fig 2:**
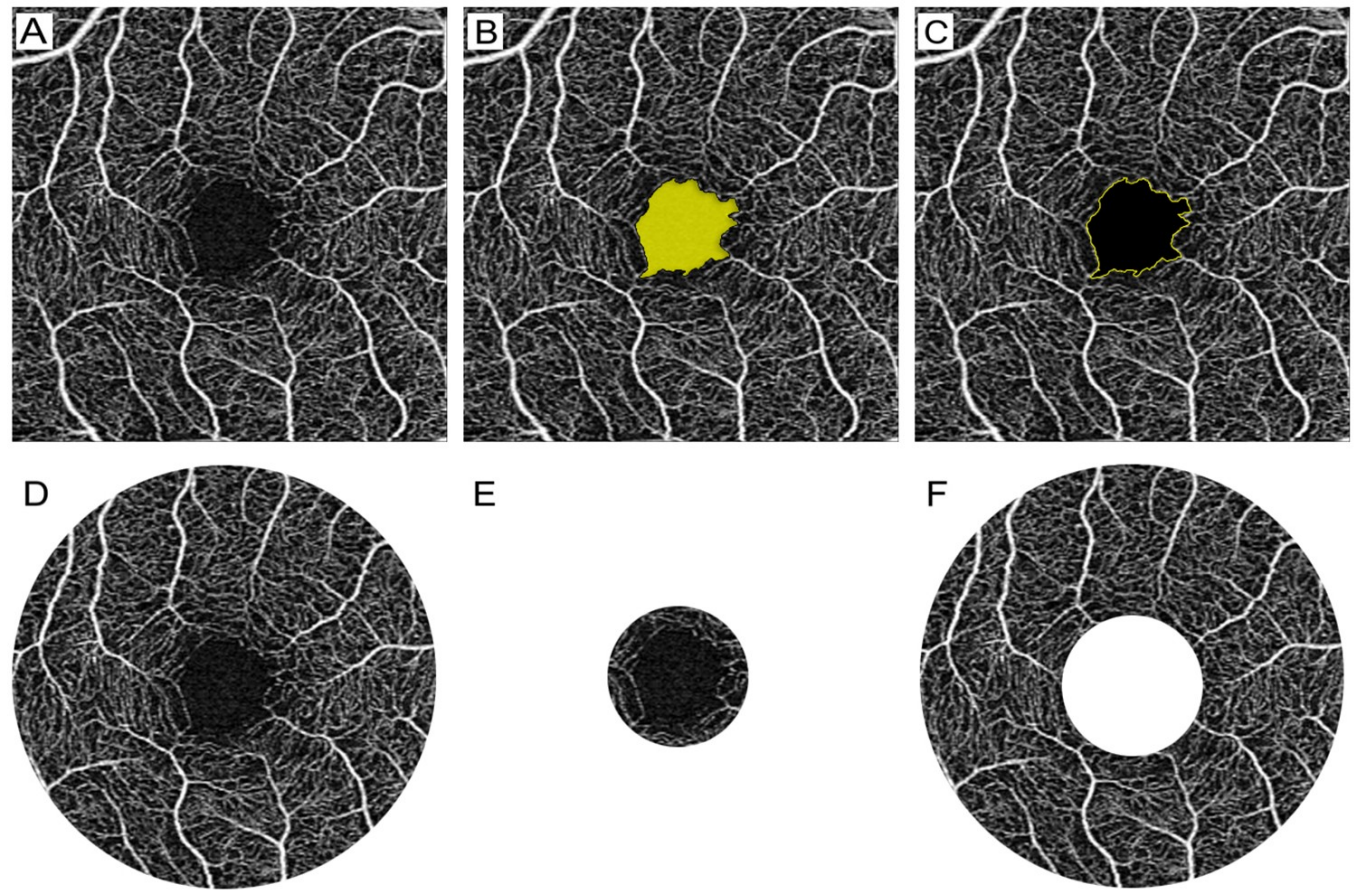
Analysis of OCTA images. (A) Original image of a 3 x 3 mm scan. (B) FAZ area is highlighted in yellow. (C) FAZ circularity is outlined in yellow. (D) The full region used to calculate PD and VLD is a circle with a 3 mm diameter. (E) The center region used to calculate PD and VLD is a circle with 1 mm diameter. (F) The inner region used to calculate PD and VLD is a ring around the center with inner and outer radii of 0.5 and 1.5 mm, respectively.

The FAZ (Figs 1A, 1B and 2A) reported by the software includes three indicators: FAZ area, FAZ perimeter and FAZ circularity. The FAZ area was the two-dimensional area of the FAZ, measured in mm^2^ (Fig 2B). The FAZ perimeter was the length of the line around the FAZ, measured in mm (Fig 2C). FAZ circularity was defined as 4π x FAZ area/(FAZ perimeter)^2^ and quantifies the degree of irregularity of the shape of the FAZ on a scale from 0 to 1.

PD (Fig 1C and 1D) was defined as the percentage of area comprised of blood vessels within the total selected area. VLD (Fig 1E and 1F) was defined as the total length of vasculature per unit area. Both PD and VLD were evaluated within specific regions: center (central circle with 1-mm diameter) (Fig 2E), inner (ring around the center with inner and outer radii of 0.5 and 1.5 mm) (Fig 2F), and full (both the central circle and the inner ring) (Fig 2D). The data reported by the software did not include an outer ring since images were limited to a 3 x 3 mm scan area.

### Statistical analysis

Qualitative data are expressed in frequencies or percentages. Quantitative data are presented as a mean ± standard deviation (SD). The normality of continuous variable distributions was tested with a Kolmogorov-Smirnov test. Quantitative variables were compared between the two groups using a t test or Mann-Whitney test if they were normally or non-normally distributed, respectively. Chi-squared testing was used to compare categorical variables. Multivariate linear regression was performed using OCTA parameters as dependent variables and the following clinical data factors as independent variables: age, sex, race, smoking history, diabetes-related parameters (duration of disease, last hemoglobin A1c, insulin use), and systemic factors (hypertension, hyperlipidemia, BMI). A p-value < 0.05 was considered to be statistically significant. Corrections for multiplicity were not made due to the high degree of correlation among the OCTA parameters. All statistics were performed using GraphPad Prism software (version 8.3.1; GraphPad Inc., San Diego, California, USA).

## Results

1099 eyes from 1099 patients with DM were included in this study and were comprised of 750 non-smokers and 349 smokers. The DM sub-type composition was as follows: type 2 DM (n = 1085), type 1 DM (n = 9), un-specified (n = 5).

Table 1 summarizes the demographic and clinical characteristics of all patients stratified by smoking history. Gender (P < 0.0001) and race (P < 0.0001) compositions were significantly different between the two groups, with a larger proportion of women in the never smoker group and a larger proportion of non-Hispanic Black and other race patients in the current smoker group. Supporting information is available online in S1 Table.

**Table 1:** Demographic and clinical characteristics stratified by smoking history.

	Total Subjects (n = 1099)	Non-Smoking Group (n = 750)	Smoking Group (n = 349)	*P* Value*
Demographics				
Age, mean ± SD, years	58.36 ± 11.22	58.39 ± 11.13	57.78 ± 11.41	0.372
Male sex	527 (48.0)	264 (35.2)	263 (75.4)	< 0.001
Race				< 0.001
Non-Hispanic White, %	85 (7.7)	46 (6.1)	39 (11.1)	
Non-Hispanic Black, %	84 (7.6)	39 (5.2)	45 (12.9)	
Hispanic or Latino, %	527 (48.0)	378 (50.4)	149 (42.7)	
Asian, %	338 (30.8)	247 (32.9)	91 (26.1)	
Other, %	65 (5.9)	40 (5.3)	25 (7.2)	
Diabetes Mellitus				
Duration, mean ± SD, years	7.57 ± 6.26	7.55 ± 6.28	7.59 ± 6.24	0.919
HbA1c, mean ± SD, %	7.70 ± 1.86	7.68 ± 1.83	7.74 ± 1.92	0.916
Insulin use, %	183 (16.7)	117 (15.6)	66 (18.9)	0.192
Comorbidities				
BMI, mean ± SD, kg/m^2^	30.88 ± 7.12	30.73 ± 6.89	31.22 ± 7.58	0.285
Hypertension, %	711 (64.7)	483 (64.4)	228 (65.3)	0.764
Hyperlipidemia, %	677 (61.6)	455 (60.7)	222 (63.6)	0.350

DR = diabetic retinopathy; SD = standard deviation; BMI = body mass index.

* For quantitative data, unpaired t-tests or Mann-Whitney tests were performed based on normality. For qualitative data, chi-squared tests were used.

Results of uncontrolled analyses are reported in Table 2. FAZ perimeter was the only OCTA parameter that was significantly different between the two groups (P = 0.033). However, FAZ area was trending towards significance (P = 0.070).

**Table 2:** OCTA parameter results stratified by smoking history.

	Non-Smoking Group (n = 750)	Smoking Group (n = 349)	*P* Value*
Foveal Avascular Zone			
FAZ area, mm^2^	0.31 ± 0.12	0.30 ± 0.13	0.07
FAZ perimeter, mm	2.51 ± 0.59	2.44 ± 0.67	0.03
FAZ circularity, %	61.4 ± 12.1	61.0 ± 10.6	0.14
Perfusion Density			
PD full, mm^2^/mm^2^, %	35.1 ± 4.0	34.8 ± 5.3	0.90
PD center, mm^2^/mm^2^, %	15.5 ± 6.2	16.1 ± 6.5	0.14
PD inner, mm^2^/mm^2^, %	37.1 ± 3.9	36.8 ± 4.9	0.68
Vessel Length Density			
VLD full, mm/mm^2^	18.80 ± 2.21	18.61 ± 2.72	0.77
VLD center, mm/mm^2^	8.69 ± 3.40	9.05 ± 3.60	0.11
VLD inner, mm/mm^2^	19.95 ± 2.48	19.68 ± 2.93	0.36

OCTA = optical coherence tomography angiography; FAZ = foveal avascular zone; PD = perfusion density; VLD = vessel length density.

* Unpaired t-tests or Mann-Whitney tests were performed for normally or non-normally distributed variables, respectively.

Select significant results from multivariate linear regression models with PD full, PD center, PD inner, VLD full, VLD center, and VLD inner as dependent variables are shown in Table 3. There were significant associations between lower VLD full (*β* = -0.31, *P* = 0.048), lower VLD inner (*β* = -0.35, *P* = 0.046) and smoking history. Larger FAZ area was associated with female sex (β = -0.05, P < 0.0001) and race. Longer FAZ perimeter was also associated with female sex (β = -0.17, P < 0.0001) and race. Higher FAZ circularity was associated with older age (β = -0.24, P < 0.0001). No significant associations between cigarette smoking and either FAZ or PD were detected. In addition, no significant associations between duration of DM or most recent HbA1c and either FAZ or PD or VLD were detected.

**Table 3:** Multivariate linear regression results of PD and VLD.

	PD full	PD center	PD inner	VLD full	VLD center	VLD inner
Age						
β	-0.10‡	-0.10‡	-0.11‡	-0.71‡	-0.06‡	-0.08‡
95% CI	-0.13 to -0.07	-0.14 to -0.06	-0.13 to -0.08	-0.85 to -0.57	-0.08 to- 0.04	-0.09 to -0.06
Male sex						
β	0.25	1.79‡	0.22	0.19	1.08‡	0.12
95% CI	-0.31 to 0.81	0.99 to 2.58	-0.31 to 0.75	-0.09 to 0.48	0.65 to 1.51	-0.20 to 0.44
Smoking status						
β	-0.41	-0.37	-0.46	-0.31*	-0.23	-0.35*
95% CI	-1.00 to 0.19	-1.21 to 0.48	-1.02 to 0.11	-0.62 to -0.00	-0.68 to 0.23	-0.69 to -0.01

PD = perfusion density; VLD = vessel length density; β = unstandardized regression coefficient; CI = confidence interval.

Smoking status is a categorical variable consisting of the following categories: Non-smoking and smoking.

The following independent variables were used in each multivariate linear regression model: Age, sex, race, smoking history, diabetes-related parameters (duration of disease, last hemoglobin A1c, insulin use), and systemic factors (hypertension, hyperlipidemia, BMI).

* = P < 0.05;

^†^ = P < 0.01;

^‡^ = P < 0.001.

## Discussion

In this study, we evaluated the association of cigarette smoking with microvascular changes in the macular region of diabetic patients without visible retinopathy using OCTA.

Microvascular abnormalities are considered to be major pathological changes in diabetic retinopathy [24]. In the early stages of diabetes, it is increasingly recognized that microvascular abnormalities can progressively affect retinal function [25]. In this study, we detected a negative association between a history of smoking and microvascular density, that is, diabetic patients with a history of smoking who had no retinopathy had lower full and inner ring region VLD values. Interestingly, no associations between smoking and the central region PD and FAZ parameters were detected. The explanation for this discrepancy is unclear; however, it may be that the extrafoveal microvasculature is more susceptible to damage from smoking. Nonetheless, these findings suggest that smoking may have a detrimental impact on the retinal microvasculature. By extension, smoking may accelerate DR-related vascular alterations at an early stage in the arc of retinopathy development and progression. These results are supported by the findings of Lee *et al*., [22] who found that current smoking was a risk factor for reduced vascular density on multivariate analysis in a small group of Korean patients who had DM for an average of three years and had no DR. The current study builds upon those findings in a large and diverse population who, on average, had diabetes for twice as long; the ability to discern the association between smoking and microvascular changes in the face of additional accumulated diabetes-related damage strengthens the attribution of these changes to smoking-related mechanisms.

Uncontrolled analysis showed that only FAZ perimeter was significantly different between the two groups in our study; however, controlled analysis revealed a larger number of significant results. This discordance can be explained by inter-group demographic differences. For example, the majority of smokers were men. Previous studies have shown that women have lower macular vascular density [26,27] and a larger FAZ [28,29] compared to men in younger patient populations. However, in contrast, studies in older adults have demonstrated that men have a lower vascular perfusion density [30,31]. Further complicating the situation is the fact that yet other demographic or clinical factors are also likely associated with vascular parameter measurements determined by OCTA. Ultimately, these considerations underscore the importance of controlling for confounding in this setting.

Interestingly, no associations were found between diabetes-related variables, such as disease duration and most recent HbA1c, and any of the studied OCTA parameters. This result is somewhat counterintuitive since these variables have been implicated in diabetic retinopathy incidence and/or progression in prior studies [3–7]. However, this result may be explained by the pre-clinical phenotype of the patients that were included in the present study. In this population, such variables may not have a significant association with microvascular changes that are only detectable with sensitive imaging modalities such as OCTA.

Older age was also found to be associated with lower PD and VLD on controlled analysis, which has been reported in previous studies [32–34]. Iafe et al. [32] reported that SCP and deep capillary plexus (DCP) vessel density decreased with age while FAZ area increased with age in normal subjects. Of note, our study found that the beta coefficients for age were larger compared to smoking history in the VLD full but not the VLD inner regression models. Our study population included diabetic patients without retinopathy, who likely differ from non-diabetic subjects in important ways. Therefore, while the contribution of older age to macular microvascular changes may be relatively large in our study, the negative association between smoking and retinal microvasculature changes should not be ignored.

Of note, FAZ characteristics may not be as useful as PD and VLD in assessing retinal microvascular changes due to the significant variation in [35,36], and ambiguous segmentation of, boundaries between the SCP and DCP [37,38]. In fact, the large variation in FAZ measurements may have contributed to the lack of significant findings with regards to the center parameters for both PD and VLD.

A key component of the prevention efforts targeting DR is appropriate screening. Early screening enables timely intervention, which can delay the development of vision loss due to the sequelae of DR in many patients. However, in resource-limited contexts, it is often not possible to screen all diabetic patients for evidence of DR [39]. In this setting, risk stratification can inform optimal referral patterns. According to our results, a history of smoking may be associated with deleterious changes in the retinal microvasculature of diabetic patients. As such, this risk factor should be considered when triaging patients from primary care clinics for a formal ophthalmic evaluation.

Limitations of this study include its cross-sectional design, which precludes any inferences regarding the predictive value of smoking on DR progression. An additional limitation is the significant demographic differences between the three studied groups. It is not unexpected that different age, sex, and race categories have different rates of smoking, and this issue was addressed with multivariate regression; however, we cannot rule out the possibility of residual confounding affecting our results. In addition, it should be noted that the imaging data used in this study was derived from the SCP in a 3 x 3 mm scan area using a spectral domain device. The relationship between smoking and microvasculature changes may be different in deeper layers of the retina, the choroid, or the non-central macula. Future studies should include analyses of the DCP, choriocapillaris, and choroid derived from a larger scan area using a swept-source device. Indeed, widefield swept-source OCTA can be particularly valuable in the detection of peripheral lesions in DR [40]. Lastly, smoking history was classified as a binary variable in our study. In future work, this exposure should be quantified (i.e. pack-years) rather than categorized in order to assess correlations including potential dose-response relationships.

## Conclusions

In conclusion, our results show that there is an association between a history of smoking and a decrease in VLD in diabetic patients without visible retinopathy. Given these results, diabetic patients with a history of smoking may benefit from higher prioritization in terms of ophthalmic screening.

## Supporting information

S1 Table Supporting data. (XLSX)
